# New system for archiving integrative structures

**DOI:** 10.1107/S2059798321010871

**Published:** 2021-11-29

**Authors:** Brinda Vallat, Benjamin Webb, Maryam Fayazi, Serban Voinea, Hongsuda Tangmunarunkit, Sai J. Ganesan, Catherine L. Lawson, John D. Westbrook, Carl Kesselman, Andrej Sali, Helen M. Berman

**Affiliations:** a RCSB PDB, Institute for Quantitative Biomedicine, Rutgers, The State University of New Jersey, Piscataway, New Jersey, USA; b Department of Bioengineering and Therapeutic Sciences, Department of Pharmaceutical Chemistry, and California Institute for Quantitative Biosciences, University of California at San Francisco, San Francisco, California, USA; c Information Sciences Institute, Viterbi School of Engineering, University of Southern California, Los Angeles, California, USA; d Department of Chemistry and Chemical Biology and Institute for Quantitative Biomedicine, Rutgers, The State University of New Jersey, Piscataway, New Jersey, USA

**Keywords:** PDB-Dev, integrative modeling, PDBx/mmCIF, IHM-dictionary, data standards

## Abstract

Structures of many complex biological assemblies are increasingly determined using integrative approaches, in which data from multiple experimental methods are combined. A standalone system, called PDB-Dev, has been developed for archiving integrative structures and making them publicly available. Here, the data standards and software tools that support PDB-Dev are described along with the new and updated components of the PDB-Dev data-collection, processing and archiving infrastructure. Following the FAIR (Findable, Accessible, Interoperable and Reusable) principles, PDB-Dev ensures that the results of integrative structure determinations are freely accessible to everyone.

## Abstract 2

A standalone system, called PDB-Dev, has been developed for archiving integrative structures and making them publicly available. The paper describes the data standards, the software tools and the various components of the PDB-Dev data-collection, processing and archiving infrastructure.

## 1. Introduction

Integrative structure determination is an approach to modeling the structures of biological systems based on information produced by multiple experimental and theoretical methods (Rout & Sali, 2019 ▸; Sali, 2021 ▸). Integrative modeling involves combining data from various experimental techniques, such as X-ray crystallography (X-ray), nuclear magnetic resonance (NMR) spectroscopy, three-dimensional electron microscopy (3DEM), small-angle solution scattering (SAS), chemical cross-linking mass spectrometry (CX-MS), Förster resonance energy transfer (FRET), electron paramagnetic resonance spectroscopy (EPR), hydrogen–deuterium exchange mass spectrometry (HDX-MS), and other biophysical and proteomics methods. The experimental data are gathered and converted into spatial restraints, and applied to experimentally determined or computationally modeled structures of molecular components. Integrative structures can be multi-scale (consisting of atomic and coarse-grained regions), multi-state (including multiple conformational states and/or heterogeneous compositions), ordered (related by time or other order) and ensembles (collection of structures, each one of which satisfies the input data). Integrative modeling techniques are typically used to model structures of macromolecular complexes that are not amenable to traditional methods of structure determination (X-ray, NMR and 3DEM).

The Protein Data Bank (PDB; Berman *et al.*, 2000 ▸; wwPDB Consortium, 2019 ▸) is the single global repository for experimentally determined structures of biological macromolecules and their complexes. Established in 1971 with seven structures (Protein Data Bank, 1971 ▸), the PDB currently contains over 180 000 structures of biological macromolecules. The PDB is the first open-access digital resource in biological sciences and is managed by the worldwide Protein Data Bank (wwPDB) organization (Berman *et al.*, 2003 ▸). The PDB archives atomic coordinates of macromolecular structures determined using X-ray, NMR and 3DEM. The PDB also archives multi-method structures that are based on one of the traditional methods (X-ray, NMR or 3DEM) used in combination with methods such as neutron diffraction or SAS. These multi-method structures contain only atomic coordinates and the different experiments are carried out on the same molecular system (for example NMR and SAS experiments carried out on the same molecular system). These multi-method structures currently archived in the PDB are a subset of integrative structures. For the PDB to be able to archive all integrative structures, including multi-scale, multi-state and ordered ensembles, changes must be made to the wwPDB OneDep data-processing pipeline (Young *et al.*, 2017 ▸).

To address the challenges presented by integrative structures, the wwPDB set up an Integrative/Hybrid Methods Task Force consisting of researchers from different fields contributing to integrative structural biology. The first meeting of the Task Force occurred in 2014 at EBI, UK. The outcomes consisted of recommendations for archiving integrative structures that were published in a white paper (Sali *et al.*, 2015 ▸). Based on these recommendations, we have created the PDB-Dev system (https://pdb-dev.wwpdb.org; Vallat *et al.*, 2018 ▸, 2019 ▸; Burley *et al.*, 2017 ▸), which provides the infrastructure for archiving integrative structures and making them publicly available following the FAIR (Findable, Accessible, Interoperable and Reusable) principles (Wilkinson *et al.*, 2016 ▸). PDB-Dev has been implemented separately from the PDB to facilitate an agile development platform. Here, we describe the different components of PDB-Dev and highlight recent improvements and updates.

## 2. The PDB-Dev archiving system

The wwPDB Integrative/Hybrid Methods Task Force provided a set of five recommendations that address the fundamental requirements for archiving integrative structures (Sali *et al.*, 2015 ▸). Our work is aligned with the Task Force recommendations and is focused on creating data standards for integrative structure determination, developing software tools that support the new data standards, and building the data pipeline for collecting, processing, archiving and distributing integrative structures. The PDB-Dev system that we have built addresses the requirements and provides the necessary infrastructure for archiving integrative structures.

### 2.1. New data standards for integrative structure determination

Data standards provide the foundation for an archive and enable data to be collected, processed and distributed in a standard form. Data standards are technical specifications that define the data and metadata to be archived and describe their syntax, semantics and logical organization. Metadata include additional descriptive information about the data, such as experimental samples and conditions, instruments and software used, authors and publications, which facilitate search, retrieval and usage of the data. In addition, data standards define metadata used for assessing and maintaining data consistency, such as data type, controlled vocabularies, boundary conditions and parent–child relationships with other data items. Data standards also provide the format for the physical encoding of the archived data.

The Protein Data Bank exchange/macromolecular Crystallographic Information Framework (PDBx/mmCIF) data representation is used by the PDB for archiving structures of macromolecules (Westbrook *et al.*, 2005 ▸; Westbrook & Fitzgerald, 2009 ▸; Westbrook, 2013 ▸). PDBx/mmCIF evolved from the CIF data representation developed by the IUCr for X-ray diffraction experiments of small molecules (Hall *et al.*, 1991 ▸). CIF was expanded to mmCIF for representing macromolecular structures determined using X-ray crystallography (Fitzgerald *et al.*, 2005 ▸), and later PDBx/mmCIF was developed as a metadata framework that allowed the representation of structures determined using NMR, 3DEM and SAS.

We have further extended the PDBx/mmCIF data representation to create data standards for describing and archiving integrative structures determined using information from multiple experimental and computational methods. The expanded data representation is embedded in a dictionary of definitions, called the IHM-dictionary (Vallat *et al.*, 2018 ▸). The IHM-dictionary is freely available from the project GitHub site (https://github.com/ihmwg/IHM-dictionary). Both PDBx/mmCIF and the IHM-dictionary allow for the creation of software that supports automated data processing.

The IHM-dictionary and PDBx/mmCIF together provide the data representation for archiving integrative structures in PDB-Dev. The representation of macromolecules and small molecules and definitions for atomic coordinates, as well as metadata information regarding authors, publications, software and macromolecular reference sequences, are all taken from PDBx/mmCIF. The expanded definitions specific for integrative modeling are captured in the IHM-dictionary and include the following.

(i) A flexible model representation for multi-scale, multi-state and ordered ensembles of integrative structures.
(ii) Definitions that support conformationally diverse ensembles and constitutionally diverse assemblies.
(iii) Descriptions of spatial restraints (including distance, angle and torsion-angle restraints) derived from many different kinds of experimental methods, such as X-ray, NMR, 3DEM, SAS, CX-MS, EPR, HDX-MS and other proteomics and bioinformatics methods.
(iv) Elucidation of starting structural models of molecular components derived from X-ray, NMR or 3DEM experiments or by computational prediction.
(v) Information regarding related experimental data available in external repositories including the PDB (wwPDB consortium, 2019 ▸), BMRB (Romero *et al.*, 2020 ▸), EMDB (Abbott *et al.*, 2018 ▸; Patwardhan & Lawson, 2016 ▸; Lawson *et al.*, 2016 ▸), EMPIAR (Iudin *et al.*, 2016 ▸), SASBDB (Kikhney *et al.*, 2020 ▸), ProteomeXchange (Deutsch *et al.*, 2017 ▸), Model Archive (Haas & Schwede, 2013 ▸; Haas *et al.*, 2013 ▸) and others, as well as data referenced via Digital Object Identifiers (DOIs).
(vi) Generic representation of modeling protocols and post-modeling analysis.

The IHM-dictionary provides definitions that support data exchange with other repositories, including definitions for database name and accession numbers for related data deposited in external repositories. Experimental data that cannot be currently archived in existing repositories can be referenced via a DOI provided by the authors.

As new structures are deposited into PDB-Dev, we work with the depositors to extend the data standards and add definitions for any new types of data/restraints used in the modeling. The data standards are extended if a submitted structure uses a new type of input restraint or modeling methodology that the current set of data standards cannot accurately represent. The existing data representation can handle most of the restraints currently used in integrative modeling investigations. This extension process, while providing flexibility, also presents an ongoing challenge for archiving integrative structures as modeling methods evolve.

### 2.2. Software support for the IHM-dictionary

Software tools developed by the wwPDB (wwPDB, 2021 ▸) and RCSB PDB (RCSB PDB, 2017*a* ▸,*b* ▸) that support PDBx/mmCIF also support the IHM-dictionary. These tools are routinely used in the PDB-Dev data pipeline discussed below. In addition to the existing dictionary tools, we have developed the *python-ihm* software library to facilitate the reading and writing of mmCIF files that are compliant with the IHM-dictionary and PDBx/mmCIF. The library is freely available from the project GitHub repository (https://github.com/ihmwg/python-ihm). Modeling software, such as *Integrative Modeling Platform* (*IMP*; Russel *et al.*, 2012 ▸) and *HADDOCK* (Dominguez *et al.*, 2003 ▸), use *python-ihm* to read and write IHM-dictionary-compliant mmCIF files that are archived in PDB-Dev. The *ChimeraX* (Goddard *et al.*, 2018 ▸) model-visualization software uses the *python-ihm* library to visualize integrative structures archived in PDB-Dev. The *Molstar* (Rose & Sehnal, 2019 ▸) web application also supports the visualization of multi-scale integrative structures represented using the IHM-dictionary.

### 2.3. PDB-Dev data pipeline

**Figure 1:**
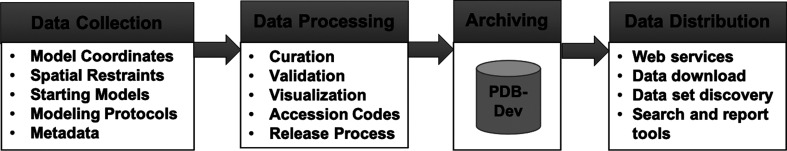
PDB-Dev archiving system. The different components of the PDB-Dev data pipeline include methods for data collection, processing, archiving and distribution.

The PDB-Dev data pipeline provides the infrastructure for collecting, processing, archiving and distributing archived data. The data standards for the PDB-Dev system are provided by the IHM-dictionary and PDBx/mmCIF. The different components of the PDB-Dev data pipeline are shown in Fig. 1 ▸ and are discussed in detail below.

#### 2.3.1. Data collection

The objective of the data-collection component of PDB-Dev is to provide a mechanism for users to deposit all relevant data associated with an integrative modeling investigation. These include three-dimensional coordinates of the modeled macromolecular complex along with the spatial restraints and starting structural models used, the modeling protocols employed and other associated metadata. The current PDB-Dev website provides a rudimentary deposition system for users to upload a complete mmCIF file that is compliant with PDBx/mmCIF and the IHM-dictionary and contains all of the required information. A compliant mmCIF file can be produced using the *python-ihm* library or directly by modeling software such as *IMP* and *HADDOCK*. Most PDB-Dev depositors, however, are unable to produce a compliant mmCIF file ready for submission and require help with the process. To help the depositors assemble all of the information that is required for archiving integrative structures, we have developed a new data-harvesting system that facilitates the collection of diverse and heterogeneous data used in integrative modeling studies. This system streamlines the data-deposition process and reduces the curation time and workload.

**Figure 2:**
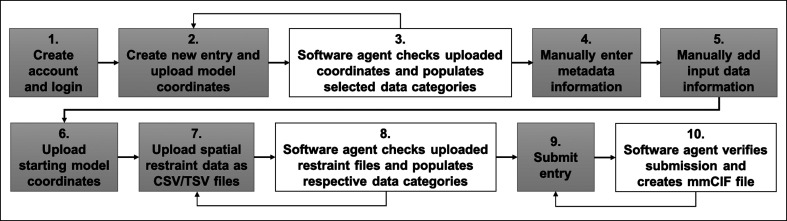
Steps involved in the PDB-Dev data-harvesting workflow. Gray boxes represent steps that are managed by the depositor/curator and white boxes represent steps carried out by software agents in the data-harvesting system. Backward arrows represent steps that allow the depositor/curator to revisit the previous step and fix any errors that are detected.

*Data-harvesting workflow*. The data-harvesting workflow for a typical deposition is broken into steps as shown in Fig. 2 ▸. We define the data and metadata associated with a single deposition to PDB-Dev as an ‘entry’. Authenticated users (step 1) can create an entry and upload the model coordinates as an mmCIF file (step 2). The user-uploaded mmCIF files are usually generated as the output of an integrative modeling study and contain model coordinates without any information regarding the starting models and spatial restraints used in the modeling or any other metadata. The uploaded mmCIF file is checked and processed by an automated software agent (step 3) to obtain additional information that can be derived from the model coordinates (for example, the number of models submitted, the components of the structural assembly and the polymeric sequence of the macromolecules modeled). This derived information is populated into the respective PDBx/mmCIF or IHM-dictionary data categories. Next, metadata regarding authors, citations, modeling protocols, reference sequence information from sequence databases [UniProt (The UniProt Consortium, 2021 ▸) and INSDC (Cochrane *et al.*, 2016 ▸)] and references to experimental data (for example, 3DEM, CX-MS, SAS and NMR) archived in other repositories [for example, PDB (wwPDB Consortium, 2019 ▸), BMRB (Romero *et al.*, 2020 ▸), EMDB (Abbott *et al.*, 2018 ▸; Patwardhan & Lawson, 2016 ▸; Lawson *et al.*, 2016 ▸), EMPIAR (Iudin *et al.*, 2016 ▸), SASBDB (Kikhney *et al.*, 2020 ▸), ProteomeXchange (Deutsch *et al.*, 2017 ▸) and Model Archive (Haas & Schwede, 2013 ▸; Haas *et al.*, 2013 ▸)] are manually added by the depositor using the web interface (steps 4 and 5). Following this, starting model coordinates and spatial restraint data are uploaded as mmCIF files and CSV/TSV files, respectively (steps 6 and 7). The automated software agent checks and processes the uploaded restraint files and populates the respective PDBx/mmCIF or IHM-dictionary data categories (step 8). Upon final submission (step 9), a complete mmCIF file is created by the system (step 10) using all of the information provided by the user including the model coordinates, spatial restraints and manually added metadata. This mmCIF file is available for users to download and for further processing by curators (Section 2.3.2).

For steps in which an automated software agent is involved (steps 3, 8 and 10), any processing errors obtained are communicated back to the user so that these errors can be fixed. Wherever data are entered manually (steps 4 and 5), based on the data definitions in PDBx/mmCIF and the IHM-dictionary, the system automatically provides access to controlled vocabulary terms where applicable and carries out data-integrity checks to enforce parent–child relationships between data items and to ensure that mandatory fields are filled and the correct data type is used (for example, text, integer, float).

The system has been designed to allow both data depositors and PDB-Dev curators to create, edit and manage the data at different stages of the data-harvesting workflow (Fig. 2 ▸). Depositors who are able to create a complete mmCIF file programmatically using *python-ihm*, *IMP* or *HADDOCK* can upload and submit such files directly using the system without going through all of the workflow steps described above.

As there are multiple steps and stakeholders (*i.e.* depositors, curators and software agents) involved in the data-harvesting workflow, the property ‘Workflow Status’ is used to communicate among different stakeholders about the status of an entry status in the workflow and the action that different stakeholders can take at any given point in time. For example, the ‘Workflow Status’ PROC indicates that it is being processed by the system, ERROR indicates that something is wrong in the entry and requires the attention of the data depositor/curator, and SUBMIT indicates that the entry has been submitted by the depositor. In addition to streamlining the complex data-submission process, the system also provides an efficient communication channel between the depositors and curators.

**Figure 3:**
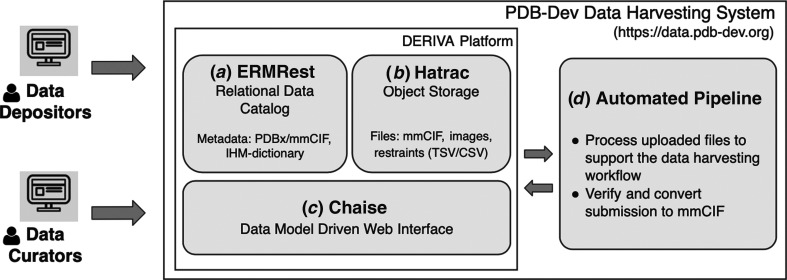
The PDB-Dev data-harvesting system consists of (*a*) a relational data catalog (ERMRest) created based on the PDBx/mmCIF and IHM-dictionary data standards, (*b*) an object-storage system (Hatrac) for user-uploaded and system-generated files, (*c*) a data model-driven web interface (Chaise) that automatically adapts its interface based on the underlying data model and (*d*) an automated pipeline to support the data-harvesting workflow. Both data depositors and curators can interact with the system using the web interface.

*Data-harvesting system*. The PDB-Dev data-harvesting system (https://data.pdb-dev.org) provides a web-based user interface that enables data depositors to create a complete and compliant mmCIF file from manually added metadata, model coordinates and restraint data associated with an integrative modeling investigation. The system has been developed using the open-source DERIVA scientific asset-management platform (https://isrd.isi.edu/deriva/; Schuler *et al.*, 2016 ▸; Bugacov *et al.*, 2017 ▸). The key components of the system (Fig. 3 ▸) are summarized below.

(i) *ERMRest data catalog and Hatrac data storage.* DERIVA provides an adaptive relational data catalog (Fig. 3 ▸ *a*) to track assets via the descriptive metadata required to describe, organize and discover these assets (Czajkowski *et al.*, 2018 ▸). Assets may be digital objects (for example, mmCIF files) or physical objects (for example, biosamples). The PDB-Dev data catalog is based on the PDBx/mmCIF data standards and the IHM-dictionary. Additionally, DERIVA provides object storage (Fig. 3 ▸ *b*) for the creation and access of digital objects including user-submitted and system-generated files. Cryptographic hashes are used to maintain data integrity and ensure that data files have not changed during upload/download operations.
(ii) *Chaise web interface*. To streamline data collection, the system provides an adaptive user interface (Fig. 3 ▸ *c*) that enables depositors/curators to create, edit or delete metadata records, upload files and update linkages between different data fields (Tangmunarunkit *et al.*, 2021 ▸). Upon submission, all data-integrity constraints (for example, mandatory fields) and data-quality controls (for example, formats of uploaded files) are checked and errors are immediately communicated to users for corrections. To help depositors/curators navigate the system, a faceted search and browse interface is provided that allows users to explore data by applying relevant search filters. A basic text-search functionality is also included, providing two complementary search approaches. Once the search results have been found, users can then browse individual data records for more detail. The DERIVA web interface is generated dynamically based on the underlying data model and is customizable, enabling us to update the data model and its presentation to fit the evolving needs of the PDB-Dev data pipeline.
(iii) *Automated pipeline*. We have developed an automated pipeline (Fig. 3 ▸ *d*) that supports the data-harvesting workflow (Fig. 2 ▸). The pipeline applies basic verification of a variety of file types, and then processes, transforms and loads the content into the data catalog ready to be linked or referred to in subsequent submission steps. Processing status and errors associated while processing individual files are communicated to the user, so that the files can be re-uploaded after the errors have been fixed. After the final submission of the entry, the pipeline aggregates all of the submitted data/metadata and generates a complete mmCIF file available for download. The automated pipeline utilizes the *python-ihm* software library to process user-uploaded files.
(iv) *Persistent IDs, data versioning and data-access methods*. The system assigns a persistent, globally unique and versioned identifier to every metadata record and digital object, enabling users to view point-in-time snapshots of metadata/data, access metadata even after the corresponding data are no longer available or the data model has changed, and reconstruct the history of changes to a metadata or data object. In addition, the system provides documented Representational State Transfer (REST) Application Programming Interfaces (APIs), high-level Python and R libraries, and command-line clients to programmatically access data.

*Access-control policies*. The data-harvesting system provides a single sign-on federated authentication service to allow users to authenticate via OAUTH2/OpenID Connect to their NIH, home institution, ORCID or Google accounts. User groups (*i.e.* depositors, curators and administrators) with different roles and privileges have been created. The system is deployed with fine-grained access-control policies that dictate who can create, modify and view the data/metadata associated with an entry throughout the data-harvesting workflow. Depositors can access all of the data/metadata associated with the entries that they have created, but cannot access entries created by other depositors. PDB-Dev curators and administrators have access to all data/metadata in the system.

*User documentation and file templates*. The website provides detailed documentation to help users understand and navigate the system. This includes documentation for account creation and access, methods to use the data-harvesting system, descriptions of the data categories in the DERIVA catalog and details of the workflow steps involved. To enable the deposition of restraint data associated with an entry, CSV file templates are provided to users. These can be downloaded and filled out to create the restraint data files for upload.

A typical deposition can be completed in a few hours if the user has all of the required information in the right format. The requirements include model coordinates (atomic or multi-scale) in mmCIF format, starting model information and coordinates (if applicable) in mmCIF format, restraint data in CSV format and metadata information such as authors, citations, modeling protocols and UniProt (The UniProt Consortium, 2021 ▸)/INSDC (Cochrane *et al.*, 2016 ▸) reference-sequence information, which can be provided through the web interface. The rate-limiting step is the collection of all of the required information prior to deposition. Additional time may be needed if the help of a curator is required to complete the deposition, which can vary on a case-to-case basis. Alternately, if the depositor submits a software-generated complete mmCIF file (for example, from *IMP*, *HADDOCK* or *python-ihm*), the deposition process can be completed within several minutes, depending upon the upload file size.

#### 2.3.2. Data processing and archiving

**Figure 4:**
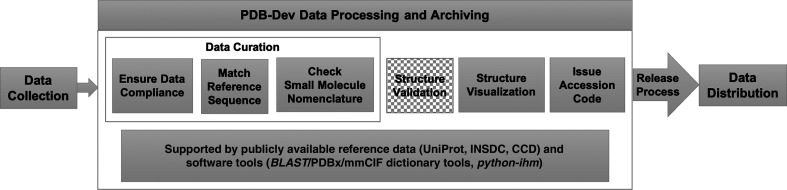
PDB-Dev data processing and archiving. Steps that have been already implemented are shown in gray boxes and steps that are under development are shown in checked boxes.

Once the data-submission process is complete, the collected data are processed in the PDB-Dev data pipeline (Fig. 4 ▸). The data-processing steps include methods for curation, validation and visualization of integrative structures. Although the specific protocols are different for integrative structures, the processing steps discussed below are aligned with the steps in the wwPDB data-processing pipeline for X-ray, NMR and 3DEM structures.

*Data curation*. The PDB-Dev data-curation steps currently involve checking for data completeness and dictionary compliance, matching the reference sequence and checking small-molecule nomenclature.

(i) *Data compliance.* The most important step in the PDB-Dev data-processing pipeline is to ensure that the required data and metadata are present in the submitted mmCIF file and that the mmCIF file is compliant with the IHM-dictionary. If the depositor provides an mmCIF file generated by *IMP*, *HADDOCK* or *python-ihm*, or if the depositor uses the PDB-Dev data-harvesting system, this step requires minimal intervention. Sometimes, users are unable to generate a compliant mmCIF file and therefore this step becomes an important and time-intensive task. We communicate with the depositors to find out how the integrative modeling was carried out, what molecular system was modeled and what input data, restraints and starting structural models were used. With the depositor-provided information in hand, we use the *python-ihm* library to generate a compliant mmCIF file. Dictionary tools created by the wwPDB (wwPDB, 2021 ▸) and RCSB PDB (RCSB PDB, 2017*a* ▸,*b* ▸) are used to check whether or not the mmCIF is compliant with PDBx/mmCIF and the IHM-dictionary.
(ii) *Reference-sequence matching.* We obtain the reference-sequence information for the macromolecules present in the submitted structure from the depositors. This information includes UniProt (The UniProt Consortium, 2021 ▸) and/or INSDC (Cochrane *et al.*, 2016 ▸) accession numbers. We then use *BLAST* (Altschul *et al.*, 1990 ▸, 1997 ▸) to align the macromolecular sequences in the submitted structure with the corresponding reference sequence in UniProt or INSDC to verify that the sequences match. Finally, the UniProt or INSDC accession numbers and details about the aligned regions are included in the appropriate PDBx/mmCIF data categories in the processed mmCIF file.
(iii) *Checking small-molecule nomenclature.* Small-molecule ligands are sometimes present in integrative structures. These are typically retained from the starting models obtained from the PDB. In this step, we ensure that any small molecule present in the submitted integrative structure is present in the Chemical Component Dictionary (CCD; Westbrook *et al.*, 2015 ▸) maintained by the wwPDB and that the nomenclature in the processed mmCIF file matches that in the CCD. Any detected errors are fixed.

*Structure validation*. At present, methods for assessing and validating integrative structures are under development based on community recommendations (Berman *et al.*, 2019 ▸). The initial version of the integrative structure-validation report will include information regarding four different categories: (i) model composition, (ii) data-quality assessment of SAS data, (iii) model-quality assessments for both atomic and coarse-grained structures and (iv) fit to the data used to build the model for SAS-based models. Data-quality assessments and evaluation of the fit to the data used to build the model are specific to the types of experimental data used to build the model and will need to be implemented separately for each data type. A fifth category, fit to the data used to validate the model, has also been proposed and is a component of the ongoing project on creating validation reports, along with the assessment of models based on other types of experimental data (such as CX-MS, 3DEM and FRET), evaluating model uncertainty and validation of conformational dynamics of ensembles. Together, these assessments will provide the user with information regarding model-quality estimates, which can be used to interpret the model. A user guide for the validation report is also being developed to help users understand the details of the different validation categories discussed above. The first version of the validation report is currently under testing and will soon be made available for all released entries in PDB-Dev.

In addition, the IHM-dictionary provides definitions for depositors to include pre-computed validation metrics that may have been used during the model-building process. These include criteria such as satisfaction/violation of cross-link distance restraints, fitting parameters for restraints derived from 3DEM, SAS and EPR data, as well as two-dimensional class-average images from electron microscopy experiments (2DEM). As validation metrics and methods are developed, we will incorporate the necessary definitions to describe validation results obtained from these methods and release a report similar to the wwPDB validation reports for X-ray, NMR and 3DEM structures.

*Structure visualization*. We have collaborated with the developers of the *Molstar* (Rose & Sehnal, 2019 ▸) and *ChimeraX* (Goddard *et al.*, 2018 ▸) molecular-visualization software to enable the visualization of multi-scale integrative structures archived in PDB-Dev. *Molstar* is a web application, whereas *ChimeraX* is a desktop application for molecular visualization. As part of the data-processing pipeline, we use both programs to visualize the integrative structures deposited in PDB-Dev. The *Molstar* application is also used on the PDB-Dev website. In addition to visualizing multi-scale models, *ChimeraX* supports the visualization of the starting structural models used and input spatial restraints derived from 2DEM, 3DEM and chemical cross-linking experiments as well as information regarding satisfied and violated cross-links. These annotations can be superimposed with the integrative structures to obtain a visual representation of how well the model fits the input data.

*Issue accession codes*. Once the structures have been processed, accession codes are issued to the depositor and updated in the mmCIF file and in the data-harvesting system. Currently, accession codes are not automatically issued upon submission because many submissions contain incomplete data. In such cases, we work with the depositors to gather all of the necessary information. With the new data-harvesting system, we expect a more streamlined submission process. We plan to implement the automated issuing of accession codes upon submission in the future. Currently, we allow depositions to be kept on hold for publication.

*Release process*. Structures are released according to depositor instructions, either upon the completion of data processing or when the associated publication is released. During release processing, the status of the entry is updated in the mmCIF file and in the data-harvesting system. Released structures are made publicly available on the PDB-Dev website.

#### 2.3.3. Data distribution

The data-distribution component of the PDB-Dev pipeline is focused on making released integrative structures findable and accessible to everyone. To enable this objective, we have built a website that provides dynamic and responsive web pages along with web services for data-set discovery and data download as well as new search and report tools.

We have developed a new search service that facilitates the retrieval of structures archived in PDB-Dev. The current implementation supports basic search using macromolecular names, accession codes, experimental data types, author names, citations, software and several other keywords. Complex queries with Boolean operators and wildcards are also supported. Search results can be further refined using filters for specific parameters such as multi-scale and multi-state ensemble attributes as well as input experimental data types. Search results can be downloaded as CSV or Excel files. The search engine has been built using the *Apache Solr* software (Apache Software Foundation, 2019 ▸) for indexing metadata. In addition to the search service, users can also browse all entries currently released in PDB-Dev and further explore the structures in detail.

We have created detailed entry pages for individual released entries that highlight important details about the structure and provide download links for each structure. The entry page for each structure includes a short summary of the structure which describes the macromolecular system being modeled, the modeling software used and the experimental restraints applied in the modeling. In addition to the description of the integrative structure, the entry pages provide information regarding the molecular assembly being modeled, the authors of the structure, citation information, software, input data and starting models used as well as related experimental and structural data residing in external repositories or accessible via DOI. The entry pages also include links to visualize the structure in the web browser using *Molstar*.

Model coordinates and related data for all released structures can be downloaded from the PDB-Dev website.

### 2.4. FAIR compliance

The PDB-Dev archiving system follows FAIR principles (Wilkinson *et al.*, 2016 ▸), including (i) web-based search interface and extensive cross-linkage of data (*findability*); (ii) globally unique persistent identifiers for all archived data and metadata (*findability*, *accessibility*, *reusability*); (iii) open interfaces for finding, retrieving, creating, updating and modifying data and metadata over the standard web HTTPS protocol (*accessibility*); (iv) interfaces for downloading metadata and data in standard formats such as mmCIF and metadata information regarding related data residing in other repositories (*interoperability*, *reusability*) and (v) a rich and standardized data model linked with standard vocabularies (*findability*, *reusability*).

### 2.5. Current status of PDB-Dev

**Figure 5:**
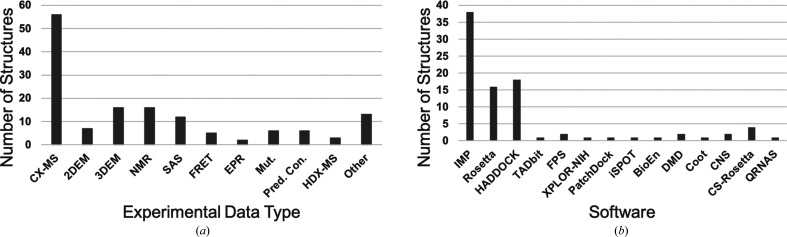
Statistics of current structures in PDB-Dev based on (*a*) the experimental data type used to obtain input spatial restraints and (*b*) the software used for modeling. The data include 66 released entries as well as 21 entries that have been processed and are kept on hold for publication as of 30 June 2021.

Development of the IHM-dictionary and the PDB-Dev data pipeline was carried out using a limited set of examples of integrative structures, including the Nup84 subcomplex (Shi *et al.*, 2014 ▸), the exosome complex (Shi *et al.*, 2015 ▸) and the mediator complex (Robinson *et al.*, 2015 ▸). Once the preliminary system had been created, we allowed users to submit new integrative structures with the expectation that availability of a variety of examples will enable further development of the IHM-dictionary as well as the supporting software tools for PDB-Dev. As of 30 June 2021, PDB-Dev contains 66 structures that have been publicly released, along with 21 structures that are on hold for publication and a handful of additional structures that are being processed. These structures have been determined using spatial restraints from different types of experimental methods such as X-ray, NMR, 3DEM, 2DEM, SAS, EPR, CX-MS, HDX-MS and others, as well as a variety of modeling software such as *IMP* (Russel *et al.*, 2012 ▸), *ROSETTA* (Leaver-Fay *et al.*, 2011 ▸), *HADDOCK* (Dominguez *et al.*, 2003 ▸), *X-PLOR-NIH* (Schwieters *et al.*, 2018 ▸), *TADbit* (Serra *et al.*, 2017 ▸), *PatchDock* (Schneidman-Duhovny *et al.*, 2005 ▸), *iSPOT* (Huang *et al.*, 2016 ▸), *BioEn* (Hummer & Köfinger, 2015 ▸), *Coot* (Emsley *et al.*, 2010 ▸), *CNS* (Brünger *et al.*, 1998 ▸), *CS-Rosetta* (Shen *et al.*, 2008 ▸), *QRNAS* (Stasiewicz *et al.*, 2019 ▸), *DMD* (Shirvanyants *et al.*, 2012 ▸) and *FPS* (Kalinin *et al.*, 2012 ▸) (Fig. 5 ▸).

**Figure 6:**
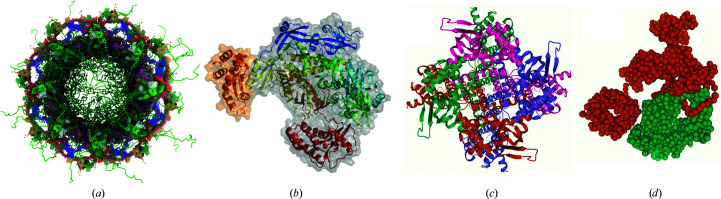
Examples of integrative structures archived in PDB-Dev. (*a*) Multi-scale structure of the nuclear pore complex from yeast (Kim *et al.*, 2018 ▸), (*b*) atomic structure of Vaccinia virus DNA polymerase catalytic subunit E9 complexed with the C-terminal region of the processivity factor component A20 (Bersch *et al.*, 2021 ▸), (*c*) one of the two conformational states obtained from multi-state modeling of the HCN voltage-gated ion channel (Dai *et al.*, 2019 ▸) and (*d*) one of the four ordered states of the human complement C3(H_2_O) from steps in a reaction pathway (Chen *et al.*, 2016 ▸).

Fig. 6 ▸ shows structures archived in PDB-Dev that highlight various features of integrative modeling, including multi-scale, multi-state and ordered ensembles, as well as the use of different modeling software and types of experimental data in the modeling. The multi-scale ensemble of the yeast nuclear pore complex (Fig. 6 ▸ *a*) was determined using IMP based on 3DEM, 2DEM, CX-MS and SAS data (Kim *et al.*, 2018 ▸), the atomic structure of Vaccinia virus DNA polymerase catalytic subunit E9 complexed with the C-terminal region of the processivity factor component A20 (Fig. 6 ▸ *b*) was obtained from *HADDOCK* using restraints derived from NMR and a titration experiment of A20 with a peptide corresponding to E9 (Bersch *et al.*, 2021 ▸), multi-state models of HCN voltage gated ion channel (Fig. 6 ▸ *c*) were determined using *ROSETTA* based on restraints from FRET data (Dai *et al.*, 2019 ▸), and the ordered states of the human complement C3(H_2_O) in a reaction pathway (Fig. 6 ▸ *d*) were obtained using *IMP *based on CX-MS restraints (Chen *et al.*, 2016 ▸).

The integrative structures archived in PDB-Dev are different from the high-resolution structures archived in the PDB in several ways. Firstly, integrative structure models can be multi-scale, multi-state, ordered, ensembles involving structures in multiple conformational and constitutional states. Secondly, integrative structures can be determined using a combination of restraints derived from a variety of experimental data types. Combining restraints from multiple experimental methods results in the use of input data at different levels of resolution, leading to structures modeled at multiple scales. Thirdly, integrative structures typically use starting structural models of components that may be obtained using experimental or computational methods. These starting models may be used as rigid bodies in integrative modeling without carrying out any additional refinement. PDB-Dev has been developed to address the specific challenges associated with archiving integrative structures, which are not addressed by the PDB.

With regard to the procedures for archiving structures of macromolecules, the current community-guided practice is as follows (Sali *et al.*, 2015 ▸). The PDB (wwPDB Consortium, 2019 ▸) archives experimental structures determined using traditional structure-determination methods such as X-ray, NMR and 3DEM. PDB-Dev archives integrative/hybrid structures determined using restraints derived from a variety of experimental data about the characterized system. The Model Archive is the repository for purely *in silico* structural models (Haas & Schwede, 2013 ▸). We await further recommendations from the community for implementing any specific deposition and archiving policies for PDB-Dev.

## 3. Summary and future perspectives

We have described how the new set of data standards, supporting software tools and the different components of the PDB-Dev system provide the foundational infrastructure for archiving and disseminating integrative structures. The PDB-Dev data pipeline currently includes methods for collecting, processing, archiving and distributing integrative structures. The PDB-Dev data-harvesting system provides an automated data-collection mechanism that helps depositors assemble all of the information required for archiving integrative structures. PDB-Dev data processing consists of data-curation steps that ensure compliance with the existing data standards as well as methods for model visualization. The PDB-Dev website includes search functionality for data-set discovery and web services for data download, thus making all released integrative structures findable and freely accessible to the public. Mechanisms for validating integrative structures based on community recommendations are currently under development and an initial version of the validation reports for released PDB-Dev structures will be made publicly available by the end of 2021. A remaining challenge is the development of comprehensive methods of validating integrative structures determined using a wide variety of experimental methods. This will require coordination with the respective experimental data communities to implement their validation protocols, in addition to other validation criteria.

The PDB-Dev system was implemented separately from the PDB in order to have an agile environment for developing the necessary mechanisms for managing integrative structures. Once the methods for collecting, processing, validating and archiving integrative structures have been fully established through PDB-Dev, our goal is to move the integrative structures archived in PDB-Dev into the PDB archive and to integrate the tools developed for PDB-Dev with the PDB infrastructure, thereby enabling the PDB to archive and disseminate all structures, including integrative structures. The integration of PDB-Dev with PDB will require coordination with the wwPDB OneDep team (Young *et al.*, 2017 ▸). One of the recommendations from the wwPDB Integrative/Hybrid Methods Task Force (Sali *et al.*, 2015 ▸) is to build a federated network of structural biology model and data archives that can interoperate with each other. The creation of such a network presents additional technical challenges such as developing shared data standards as well as methods for federated data exchange.

Finally, as the science evolves, the data standards and associated data-processing mechanisms will need to be adapted so that the archiving requirements of the structural biology community are met. Developing a rigorous standardized process for the deposition, curation, validation, archival and dissemination of integrative structures will empower the scientific community to tackle very large structure-determination challenges, such as elucidation of the three-dimensional structures of complete genomes (Hua *et al.*, 2018 ▸; Joseph *et al.*, 2017 ▸; Di Stefano *et al.*, 2021 ▸) and even the creation of a spatiotemporal model of an entire cell (Singla *et al.*, 2018 ▸).
